# Typhoid Fever

**Published:** 1859-01

**Authors:** B. O. Reynolds

**Affiliations:** Elk Horn, Wis.


ARTICLE VI.

### TYPHOID FEVER.

BY B. O. REYNOLDS, M. D.

Typhoid fever has prevailed in this vicinity for the last three months to a considerable extent, and quite a number of deaths have occurred from this cause. All the cases, however, that have come under my immediate observation have been of an ordinarily mild character. Indeed, it is my opinion, based upon close observation, that nearly all the cases of this disease occurring in the country, or in an otherwise healthy locality, would continue mild were it not for the common practice of too many physicians, especially those of ancient origin, to dose freely with calomel and other active cathartics, thereby reducing the plasticity of the blood, as well as directly irritating the intestinal glands, which, I believe, are universally spongy or inflamed in this form of fever.

Within the last three years I have treated thirty well-marked cases of this disease, and all, with two exceptions, without the use of mercurials in any form, and also without cathartics, using in their stead, when necessary, simple laxatives or enemas.

The exceptional cases referred to presented decided biliary derangements, and a few doses of hydr. cum creta seemed to have a beneficial effect.

Unless contra-indicated by local complications, I give tonics throughout the entire course, and early resort to the turpentine emulsion, aided by a judicious administration of well-salted animal broths and other supporting diet. If I could use but one remedy in the treatment of this disease, I should greatly prefer the turpentine to any other.

I have for several years closely watched the result of the calomel treatment as practised by our old fogyish physicians (who use mercurials invariably in all fevers), and must say that the mortality has been considerable, and many who have finally recovered narrowly escaped death.

All the cases that I have been called to treat from the commencement of the attack, and before any medicine had been given, have not only recovered, but the disease, after the first six or eight days, generally assumed a mild character, the patients getting about in from one to four weeks. Either the cases that have come immediately under my observation and treatment have all been miraculously mild, or else the treatment had something to do in mitigating the disease.

Again, I have frequently been called to see patients that had been copiously purged, either professionally or, by what is a common practice with many persons when attacked with disease, by taking large portions of patent pills, which generally are composed largely of aloes or other drastic cathartics, who very soon presented the disease in its worst form, and some have died about the third week, while others barely escaped, and only recovered after an illness of from five to eight weeks.

I do not wish to be understood to say that this or any other fever may not be attended with alarming symptoms, and even prove fatal under any treatment. Undoubtedly such cases do occasionally occur; but what I would impress upon the minds of your readers is, that in our treatment we should carefully avoid anything that would tend to impair the already much depressed vital forces of the system, which we have every reason to believe is the true condition in this disease.

I have been induced to say this much from the fact that I have so often been surprised to see some of my senior brethren not only saturating their typhoid patients with mercurials, but also administering freely hydragogue cathartics, even in advanced stages, when there was evidence of great general prostration.

Our county medical society holds regular meetings, yet it is a painful fact that the interest taken by the profession throughout the north-west in these societies is not what their importance demands. At our last meeting the following resolution was unanimously adopted, viz: That we believe a very large proportion of human misery, including poverty, disease and crime, is induced by the use of intoxicating drinks; and the most perfect health is compatible with total abstinence from all such drinks as beverages, whether in the form of distilled or fermented spirits; and we believe that persons accustomed to the use of such drinks may, with perfect safety, discontinue them entirely; that total and universal abstinence from alcoholic beverages of all kinds would greatly contribute to the health, the prosperity, the morality, the longevity, and the happiness of the human race.

I believe if this or a similar resolution was promulgated by every physician, endorsed and made public by every local medical society, that much in this way might be done to eradicate that monster disease, *intemperance.*

ELK HORN, WIS., May, 1858.

